# Genetic Variation of *Migration Inhibitory Factor* Gene rs2070766 Is Associated With Acute Coronary Syndromes in Chinese Population

**DOI:** 10.3389/fgene.2021.750975

**Published:** 2022-01-03

**Authors:** Jin-Yu Zhang, Qian Zhao, Fen Liu, De-Yang Li, Li Men, Jun-Yi Luo, Ling Zhao, Xiao-Mei Li, Xiao-Ming Gao, Yi-Ning Yang

**Affiliations:** ^1^ State Key Laboratory of Pathogenesis, Prevention and Treatment of High Incidence Diseases in Central Asia, Department of Cardiology, First Affiliated Hospital of Xinjiang Medical University, Urumqi, China; ^2^ Rehabilitation Department of First Affiliated Hospital of Xinjiang Medical University, Urumqi, China; ^3^ Xinjiang Key Laboratory of Cardiovascular Disease Research, Clinical Medical Research Institute of Xinjiang Medical University, Urumqi, China; ^4^ Xinjiang Key Laboratory of Medical Animal Model Research, Clinical Medical Research Institute of Xinjiang Medical University, Urumqi, China; ^5^ People’s Hospital of Xinjiang Uygur Autonomous Region, Urumqi, China

**Keywords:** macrophage migration inhibitory factor, acute coronary syndromes, genetic variant, nomogram, major adverse cardiovascular events

## Abstract

Genetic variation of macrophage migration inhibitory factor (*MIF*) gene has been linked to coronary artery disease. We investigated an association between the polymorphism of *MIF* gene rs2070766 and acute coronary syndromes (ACS) and the predictive value of *MIF* gene variation in clinical outcomes. This study involved in 963 ACS patients and 932 control subjects from a Chinese population. All participants were genotyped for the single nucleotide polymorphism (SNP) of *MIF* gene rs2070766 using SNPscan™. A nomogram model using *MIF* genetic variation and clinical variables was established to predict risk of ACS. Major adverse cardiovascular events (MACE) were monitored during a follow-up period. The frequency of rs2070766 GG genotype was higher in ACS patients than in control subjects (6.2 vs 3.8%, *p* = 0.034). Multivariate logistic regression analysis revealed that individuals with mutant GG genotype had a 1.7-fold higher risk of ACS compared with individuals with CC or CG genotypes. Using *MIF* rs2070766 genotypes and clinical factors, we developed a nomogram model to predict risk of ACS. The nomogram model had a good discrimination with an area under the curve of 0.781 (95% CI: 0.759–0.804), concordance index of 0.784 (95% CI: 0.762–0.806) and well-fitted calibration. During the follow-up period of 25 months, Kaplan-Meier curves demonstrated that ACS patients carrying GG phenotype developed more MACE compared to CC or CG carriers (*p* < 0.05). GG genotype of *MIF* gene rs2070766 was associated with a higher risk of ACS in a Chinese population. The GG genotype carriers in ACS patients had worse clinical outcomes compared with those carrying CC or CG genotype. Together with rs2070766 genetic variant of *MIF* gene, we established a novel nomogram model that can provide individualized prediction for ACS.

## Introduction

Acute coronary syndromes (ACS), an acute form of coronary artery disease (CAD), is the leading cause of death, making a worldwide health concern (Hyde et al., 2020). ACS describes a spectrum of clinical manifestations including unstable angina (UA), ST-segment elevation myocardial infarction (STEMI), and non-STEMI (NSTEMI) (Mason et al., 2018). As a complex disease, both genetic and environmental factors contribute to ACS susceptibility (Roberts and Campillo, 2018). Advances in exome-wide association study have provided insights into several candidate genes and pathways that contribute to ACS (Zheng et al., 2020). Although the technology of percutaneous coronary intervention (PCI) and drug therapy has been constantly improved, ACS is still characterized by high morbidity and unsatisfactory prognosis. Therefore, the molecular mechanisms involved in the initiation and development of ACS still need to be explored, which will contribute to better management for ACS patients.

Ample evidences suggest that ACS is triggered by an inflammatory response and plaque destabilization as indicated by increased inflammatory processes at the site of intimal rupture and elevated circulating levels of inflammatory biomarkers during the event (Gresele et al., 2011). Macrophage migration inhibitory factor (MIF) is a pro-inflammatory cytokine expressed in various mammalian cells (Muller et al., 2012). Numerous experimental and clinical studies have identified the involvement of MIF in the progression of vascular atherosclerosis (Burger-Kentischer et al., 2002; Pan et al., 2004; Sinitski et al., 2019). Notably, MIF has also been reported to be associated with plaque instability (Schmeisser et al., 2005). A number of previous clinical studies examined the predictive value of the circulating MIF levels for future cardiac events. Boekholdt et al. reported that the relation between MIF and the risk of MI or death due to CAD in adults without a history of MI or stroke was not very strong. However, MIF is involved in the inflammatory processes that underlie atherosclerosis (Boekholdt et al., 2004). Makino et al. demonstrated that the high MIF level was an independent risk factor for future coronary events in CAD patients with type 2 diabetes mellitus (DM) (Makino et al., 2010). Later, our experimental and clinical findings indicate that a single MIF assay at admission could be a useful biomarker for early prediction of final infarct size and extent of cardiac remodeling (Chan et al., 2013). A higher admission MIF level is an independent predictor for in-hospital mortality and long-term major adverse cardio-and/or cerebrovascular events (MACCE) in STEMI patients who underwent PCI (Zhao et al., 2019). Hence, MIF might be a potential biomarker to predict the risk and severity of CAD.

The *MIF* gene, located at chromosome 22q 11.2, is a small gene consisting of three exons that are 205, 173 and 183 base pairs in length (Lan et al., 2013). The promoter region *MIF* gene focused on the -794 (CATT)_5–8_ microsatellite (rs5844572) and the -173 G/C (rs755622) polymorphisms have been extensively studied for its association with CAD (Lehmann et al., 2006; Tereshchenko et al., 2009; Valdes-Alvarado et al., 2014; Luo et al., 2016). Gene reporter assays showed that an increased transcription of *MIF* gene rs5844572 with the 5-repeat allele led to a low expression of MIF, while increase of the 6-, 7-, and 8-repeat alleles led to a correspondingly higher expression of MIF (Baugh et al., 2002; Radstake et al., 2005). An association of 6/7 genotype of the *MIF* -794 (CATT)_5–8_ polymorphism with susceptibility to ACS has been observed in Mexican population (Valdes-Alvarado et al., 2014). A second *MIF* gene promoter polymorphism comprises a G-to-C single nucleotide polymorphism (SNP) at position of -173 (rs755622) has also been broadly investigated for its association with the severity of CAD (Lehmann et al., 2006; Tereshchenko et al., 2009; Luo et al., 2016; Coban et al., 2019). Our previous study showed that *MIF* gene rs755622 CC genotype carriers had the highest plasma levels of MIF than CG and GG genotype carriers in ACS patients (Du et al., 2020). A recent meta-analysis also demonstrated plenty evidences for the associations between *MIF* -173C/G and CAD susceptibility in different populations (Li et al., 2020).

Except SNPs in the promoter of *MIF* gene, variation in other regions i.e. coding region or intron may also have a potential influence in the risk of CAD. An association between the variation of *MIF* gene rs2070766 in the intron and acute lung injury has been found (Gao et al., 2007). So far, there is no study focusing on the intron polymorphism of *MIF* gene in relation to CAD. The purpose of this study is to investigate whether the variant rs2070766 located in the intron of *MIF* gene is associated with susceptibility of ACS in a Chinese population. We also assess the value of *MIF* gene rs2070766 polymorphism in predicting clinical outcomes in ACS patients.

## Materials and Methods

### Ethics Approval of Study Protocol

Written informed consents were obtained from all participants. The study protocol was conducted according to the standards of the Declaration of Helsinki, and the study was approved by the Ethics Committee of the First Affiliated Hospital of Xinjiang Medical University.

### Study Design and Participants

**FIGURE 1:**
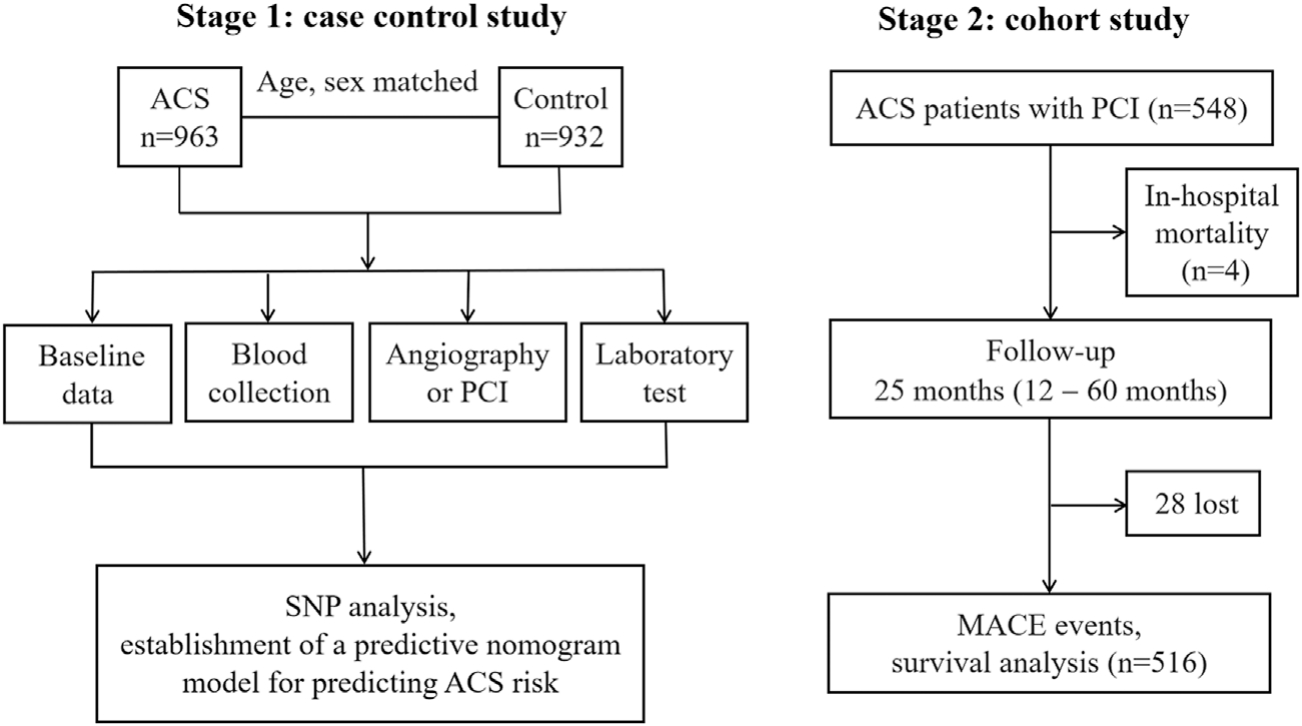
The flow chart of study design. PCI, percutaneous coronary intervention; SNP, single nucleotide polymorphism; ACS, acute coronary syndromes; MACE, major adverse cardiovascular events.

This study was divided into two stages (Figure 1). *First,* a single-center hospital-based case-control study including ACS patients and control subjects were recruited from the First Affiliated Hospital of Xinjiang Medical University from January 2014 to December 2017. ACS patients were diagnosed and classified according to the criteria of the American College of Cardiology including UA, STEMI and NSTEMI (Cannon et al., 2013). The diagnostic triad included clinical symptoms, electrocardiogram changes and alterations in cardiac biomarkers (creatine kinase; creatine kinase-MB isoenzyme and troponine I). All ACS patients underwent coronary angiography to identify the culprit-vessel, i.e., ≥ 50% luminal stenosis in at least one coronary artery or major branch segments. The findings of coronary angiography were interpreted by at least two experienced cardiologists. All ACS patients received 300 mg of aspirin and a 300 mg loading dose of clopidogrel at admission and 70 U/kg of standard intravenous heparin before the PCI. After PCI, all patients received dual antiplatelet therapy: 100 mg aspirin daily, and 75 mg clopidogrel daily for at least 1 year. Other cardiac medications were given at the discretion of the attending physicians. During the same period, age and sex matched control participants who suffered from atypical chest pain and admitted into hospital but with normal coronary angiogram and showed no clinical evidence of ischemic heart disease were also recruited. Therefore these control subjects were not healthy individuals, indicating that the control group was also exposed to the same risk factors of ACS. As per past medical history, individuals who had regional wall motion abnormalities, valvular abnormalities in echocardiograms, previous MI received coronary artery bypass grafting, previous heart transplants, chronic inflammation established by clinical, laboratory, or image investigations, malignant tumors, type 1 diabetes, chronic kidney disease (stages 3–5, estimated glomerular filtration rate <60 ml/min/1.73 m^2^), or liver enzyme elevation exceeding three times the upper limit of normal were excluded. *Second*, we followed up ACS patients who received PCI, it was a single-center, prospective cohort study designed to assess influence of different genotypes of *MIF* gene rs2070766 on long-term prognosis of ACS.

### Blood Collection and Laboratory Test

Venous blood samples were drawn at the catheter laboratory before angiography from ACS patients and from control subjects during medical examination. Full blood examination and biochemical assays were performed using the commercially available automated platform in the Central Laboratory of the First Affiliated Hospital of Xinjiang Medical University. These tests included white blood cell count (WBC), platelet (PLT), urea nitrogen (BUN), creatinine (CR), triglyceride (TG), total cholesterol (TC), high-or low-density lipoprotein-cholesterol (HDL-C, LDL-C).

### Deoxyribonucleic Acid Extraction

Deoxyribonucleic acid (DNA) extraction from venous blood was performed after laboratory test. Venous blood samples with ethylene diamine tetra acetic acid (EDTA) as the anticoagulant were centrifuged at 4,000 × *g* for 5 min to separate plasma and blood cells. DNA was extracted from the peripheral leukocytes using a whole-blood genome extraction kit (Beijing Bioteke Corporation, China) following the manufacturer’s instruction (Adi et al., 2020). DNA samples were stored at −80°C for genotyping.

### Genotyping of Migration Inhibitory Factor Gene

Sample DNA (10 ng) were amplified by polymerase chain reaction (PCR) according to the manufacturer’s recommendations. The SNP genotyping work was performed using a custom-by-design 50-Plex SNPscan™ Kit (Genesky Biotechnologies Inc., Shanghai, China). For quality control, repeated analyses were done for 4% of randomly selected samples with high genotyping quality. Variants of *MIF* gene rs2070766 were classified into three genotypes, CC, CG, and GG. The dominant model is defined as the wild homozygous genotype (CC) versus heterozygous genotype (CG) plus mutant homozygous genotype (GG), a recessive model is defined as mutant homozygous genotype (GG) versus wild homozygous genotype (CC) plus heterozygous genotype (CG) and an additive model is defined as heterozygous genotype (CG) versus wild homozygous genotype (CC) plus mutant homozygous genotype (GG).

### Definition of Cardiovascular Risk Factors

Body mass index (BMI) was calculated by dividing body weight (in kg) by the height in meters squared to determine the risk of obesity. Persons reporting regular tobacco use in the previous 6 months were considered as current smokers. Drinker was defined as consuming 100 g at least once alcoholic beverage per week in the past month. Hypertension was defined as systolic BP (SBP) 140 mmHg, and/or diastolic BP (DBP) 90 mmHg, and/or use of antihypertensive medicine within 2 weeks, based on 2018 ESC/ESH Guideline (Heizhati et al., 2020). DM was defined as fasting plasma glucose levels ≥7.0 mmol/L (126 mg/dl), glucose levels ≥11.1 mmol/L (200 mg/dl) 2 h after the administration of a 75 g oral glucose load, a history of diabetes or patients with a history of anti-diabetic medication use. Concentrations of TG ≥ 2.26 mmol/L (200 mg/dl), TC ≥ 6.22 mmol/L (240 mg/dl), HDL-C < 1.04 mmol/L (40 mg/dl) and LDL-C ≥ 4.14 mmol/L (160 mg/dl) were defined as hypertriglyceridemia, hypercholesterolemia, hypo-HDL-C and hyper-LDL-C respectively. Dyslipidemia was defined as anyone of the four lipids abnormalities above or self-reported use of antihyperlipidemic medication (Pan et al., 2013).

### Study Endpoints During the Follow-Up Period

During hospitalization and after discharge, major adverse cardiovascular events (MACE) (Zhao et al., 2019) including re-hospitalization owing to recurrent angina, re-hospitalization owing to heart failure, target lesion revascularization, cardiac death, non-fatal MI and stent thrombosis were monitored as the study endpoint. Follow-up protocol included phone interview, outpatient visiting and in-hospital clinical records of patients who were rehospitalized. Information of deceased patients was obtained from hospital records or phone contact with relatives of the patients. The frequency of contact was every 3 months for the first year, later every 6 months for later follow-up period. During the follow-up duration, an independent group of clinical physicians carefully checked and verified all events. To obtain high-quality data, all attending investigators were trained and data entry was performed by two investigators.

### Statistical Analyses

Data were collected using Epidata 3.1 (Odense, Denmark) and double checked. Analyses were carried out using Stata 15.0 software (Stata Corp LP, College Station, TX, United States). Continuous variables with a Gaussian distribution are presented as mean ± standard deviation (SD), and those with a non-Gaussian distribution are presented as median values with corresponding 25th to 75th percentiles. The differences between groups were evaluated using Student’s unpaired *t* test or the Mann-Whitney rank test. Categorical variables were expressed as numbers or frequencies and the difference between groups was detected by Chi-square test. Chi-square test was also used to calculate Hardy-Weinberg equilibrium of the frequencies of genotype between ACS and control subjects. Logistic regression analyses with effect ratios (odds ratio [OR] and 95% confidence interval [CI]) were used to assess the contribution of the major risk factors. Kaplan-Meier plots were generated and the log-rank test was used to compare the survive curve among the different genotype carriers. *p* value < 0.05 was considered statistically significant.

By applying multivariate logistic regression, we established an ACS risk predictive model of nomogram. The scoring system in the nomogram was generated by the RMS (Regression Modeling Strategies) package (available at the website: https://cran.r-project.org/web/packages/rms/index.html) based on R-language (version 3.5.3) (available at the website: https://www.r-project.org) (Figure 3A). The nomogram was expressed as the total score (point) for each nodule (individual variable) (Zhou et al., 2020). The total points scale is added including all independent variables which are converted to predicted probabilities. In detail, for categorical variables (yes/no, GG/CC + CG genotypes) and continuous variables, according to this calculate method, the length of individual horizontal line represents a degree of its contribution to the ACS risk. The vertical points at the end of horizontal line corresponding to a specific value on the point scale (top) (Figure 3A). The nomogram model was evaluated from three aspects: discrimination ability (Figure 4A), calibration ability (Figure 4B), and clinical effectiveness (Figure 4C). *First*, receiver operation characteristic curve (ROC) was used to evaluate the discrimination. The value of area under curve (AUC) exists between 0.5 and 1. A value of the AUC closer to 1 indicates a good performance of the predictive model (Harrell et al., 1982). Discrimination was quantified using Harrell’s concordance index (C-index), in which an absolute value close to 1 indicated that the model had strong predictive ability. The C-index is equivalent to the area under the receiver operating characteristic curve, and it is used to measure how well a model predicts the disease risk. C-index > 0.7 was considered to have excellent discrimination (Kim et al., 2018) (Figure 4A). *Second*, calibration plots were developed to assess the predictive accuracy and agreement between predicted and observed severity. The 45° diagonal line in the plot indicates a perfect calibrated curve which has a best predictive capability for the actual risk of disease. The calibration capability was evaluated through the calibration chart and the Hosmer-Lemeshow test (Kramer and Zimmerman, 2007) (Figure 4B). If the smaller the Chi square value of the statistics is, the larger the corresponding *p* value is, the better the calibration of the predictive model will be. If the test results show statistical significance (*p* < 0.05), indicating a certain difference between the predicted value of the model and the actual observed value, and the model calibration is poor (Kramer and Zimmerman, 2007). The nomogram was further internally validated by the bootstrap method with 1,000 resamples to measure the AUC value, C-index, and calibration curve (Schomaker and Heumann, 2018). *Third,* a decision curve analysis (DCA) was used to evaluate the clinical usefulness of the nomogram based on its net benefits at different threshold probabilities (Kerr et al., 2016) (Figure 4C). The net benefit was calculated by subtracting the proportion of patients with false-positive results from the proportion of patients with true-positive results and by weighing the relative risk of an intervention compared with the adverse effects of an unnecessary intervention.

## Results

### Characteristics of Study Participants

Demographic and clinical characteristics of the study participants are shown in Table 1. In total, 963 ACS patients (68.2% men) and 932 control subjects (65.2% men) were recruited in the present study. No significant differences were observed in age, sex, BMI, drinking and plasma level of BUN between the two groups. Nevertheless, WBC, PLT and plasma levels of CR, TG, TC and LDL-C were higher in ACS patients than that in controls (all *p < 0.05*). In addition, the plasma level of HDL-C was higher in controls compared with ACS patients both in male and female (*p < 0.05*). The prevalence of smoking, hypertension, diabetes and dyslipidemia were greater in ACS patients than in controls (all *p < 0.05*). To explore the gender difference, male and female participants in both ACS and control groups were separated. In male participants, no significant differences were observed in age, BMI, plasma levels of BUN, CR and TG and prevalence of drinking and hypertension between the two groups. Whilst, WBC, PLT, plasma levels of TC, LDL-C and the prevalence of smoking, diabetes and dyslipidemia were notably higher in ACS than in control males (all *p < 0.05*). In female participants, age, BMI and plasma levels of BUN and LDL-C were comparable between the two groups. There were significant differences in WBC, PLT, plasma levels of CR, TG, TC and HDL-C and the prevalence of hypertension, diabetes and dyslipidemia between ACS and control females (all *p < 0.05*).

**TABLE 1:** Demographic and clinical characteristics of the study population.

	Total			Male			Female		
	ACS	Control	*p* Value	ACS	Control	*p* Value	ACS	Control	*p* Value
Number, n	963	932		657	608		306	324	
Age (years)	56.1 ± 10.2	55.8 ± 9.2	0.621	53.4 ± 9.4	52.6 ± 9.3	0.117	61.7 ± 9.4	61.9 ± 5.0	0.732
Male, n (%)	657 (68.2%)	608 (65.2%)	0.167	-	-	-	-	-	-
BMI (kg/m^2)^	25.9 ± 3.4	26.3 ± 3.7	0.079	26.0 ± 3.3	26.5 ± 3.5	0.097	25.4 ± 3.7	26.0 ± 4.0	0.184
Smoking, n (%)	430 (44.8)	338 (36.3)	<0.001	421 (64.3)	338 (55.6)	0.002	9 (2.9)	0 (0.0)	-
Drinking, n (%)	291 (30.3)	273 (29.3)	0.638	284 (43.4)	273 (44.9)	0.581	7 (2.3)	0 (0.0)	-
Hypertension, n (%)	476 (49.4)	404 (43.4)	0.008	280 (42.6)	261 (42.9)	0.911	196 (64.1)	143 (44.1)	<0.001
Diabetes, n (%)	251 (26.1)	112 (12.0)	<0.001	150 (22.8)	62 (10.2)	<0.001	101 (33.0)	50 (15.4)	<0.001
WBC, 10^9^/L	9.42 ± 3.56	6.81 ± 2.12	<0.001	10.07 ± 3.69	7.01 ± 1.98	<0.001	8.05 ± 2.81	6.46 ± 2.32	<0.001
PLT, 10^9^/L	233.95 ± 65.01	217.09 ± 56.21	<0.001	232.68 ± 65.55	212.73 ± 52.62	<0.001	236.68 ± 63.86	225.05 ± 61.54	0.024
BUN (mmol/L)	5.48 ± 1.88	5.43 ± 1.55	0.508	5.57 ± 1.86	5.55 ± 1.56	0.868	5.29 ± 1.90	5.19 ± 1.50	0.480
CR (umol/L)	73.36 ± 20.05	71.51 ± 16.85	0.032	78.16 ± 19.71	77.54 ± 15.07	0.538	62.81 ± 16.44	59.96 ± 13.80	0.022
TG (mmol/L)	1.63 (1.11–2.44)	1.56 (1.04–2.26)	0.025	1.63 (1.09–2.50)	1.65 (1.06–2.42)	0.759	1.64 (1.12–2.42)	1.39 (1.02–1.96)	0.001
TC (mmol/L)	4.47 ± 1.29	4.16 ± 0.94	<0.001	4.46 ± 1.29	4.11 ± 0.93	<0.001	4.49 ± 1.29	4.27 ± 0.95	0.021
HDL-C (mmol/L)	0.95 (0.80–1.14)	1.06 (0.87–1.27)	<0.001	0.91 (0.78–1.08)	0.98 (0.82–1.19)	<0.001	1.07 (0.90–1.29)	1.20 (0.99–1.39)	<0.001
LDL-C (mmol/L)	2.75 (2.17–3.40)	2.58 (2.05–3.16)	<0.001	2.76 (2.23–3.41)	2.58 (2.04–3.13)	<0.001	2.74 (2.05–3.37)	2.59 (2.10–3.21)	0.317
Dyslipidemia, n (%)	653 (73.9)	494 (57.9)	<0.001	483 (79.1)	374 (66.6)	<0.001	170 (62.3)	120 (41.1)	<0.001

Continuous variables are expressed as mean ± SD, or median (25th-75th percentiles). Categorical variables are expressed as number and percentage. Abbreviations: ACS, acute coronary syndromes; BMI, body mass index; WBC, white blood cells; PLT, platelet; BUN, blood urea nitrogen; CR, creatinine; TG, triglycerides; TC, total cholesterol; HDL-C, high-density lipoprotein cholesterol; LDL-C, low-density lipoprotein cholesterol.

### The Frequency of Mutant GG Genotype Were Significantly Higher in Acute Coronary Syndromes Patients

Distribution of *MIF* gene rs2070766 variation was in Hardy-Weinberg equilibrium in both the ACS and control groups (data not shown). The frequencies of genotypes and alleles of *MIF* gene rs2070766 are presented in Table 2. The results showed that the mutant GG genotype (*p =* 0.034 for all participants) and recessive model (GG vs CC + CG) in all participants (*p =* 0.019) and in females (*p =* 0.028) were more frequent in the ACS patients than in the control subjects. While, there was no significant difference in distribution of dominant and additive models and alleles in rs2070766 between ACS and control groups (all *p >* 0.05).

**TABLE 2:** Distribution of genetic variation of *MIF* gene rs2070766 in the study population.

		Total			Male			Female		
		ACS, n (%)	Control, n (%)	*p*	ACS, n (%)	Control, n (%)	*p*	ACS, n (%)	Control, n (%)	*p*
Genotype	CC	586 (60.9)	559 (60.0)	0.034	404 (61.5)	361 (59.4)	0.214	182 (59.5)	198 (61.1)	0.087
	CG	317 (32.9)	337 (36.2)		218 (33.2)	224 (36.8)		99 (32.4)	113 (34.9)	
	GG	60 (6.2)	36 (3.8)		35 (5.3)	23 (3.8)		25 (8.2)	13 (4.0)	
Dominant model	CC	586 (60.9)	559 (60.0)	0.698	404 (61.5)	361 (59.4)	0.442	182 (59.5)	198 (61.1)	0.675
	GG + CG	377 (39.1)	373 (40.0)		253 (38.5)	247 (40.6)		124 (40.5)	126 (38.9)	
Recessive model	GG	60 (6.2)	36 (3.8)	0.019	35 (5.3)	23 (3.8)	0.189	25 (8.2)	13 (4.0)	0.028
	CC + CG	903 (93.8)	896 (96.2)		622 (94.7)	585 (96.2)		281 (91.8)	311 (96.0)	
Additive model	CG	317 (32.9)	337 (36.2)	0.138	218 (33.2)	224 (36.8)	0.172	99 (32.4)	113 (34.9)	0.503
	CC + GG	646 (67.1)	595 (63.8)		439 (66.8)	384 (63.2)		207 (67.6)	211 (65.1)	
Allele	C	1,489 (77.3)	1,455 (78.1)	0.581	1,026 (78.1)	946 (77.8)	0.862	463 (75.7)	509 (78.6)	0.221
	G	437 (22.7)	409 (21.9)		288 (21.9)	270 (22.2)		149 (24.3)	139 (21.4)	

ACS, acute coronary syndromes.

### Migration Inhibitory Factor Gene Mutant GG Genotype Was Associated With a Higher Risk of Acute Coronary Syndromes

Univariate regression analysis showed that the GG genotype in rs2070766 was a risk factor for ACS (Table 3, GG genotype vs CC + CG genotypes, OR 1.654, 95% CI: 1.083–2.526; *p* = 0.020). Moreover, smoking, hypertension, diabetes, WBC, TC and LDL-C were risk factors for ACS. HDL-C was a protect factor for ACS. Multivariate logistic regression analysis revealed five independent factors for ACS: diabetes, mutant GG genotype, WBC, TC and HDL-C. After adjustments of smoking, hypertension, diabetes, WBC, TC, HDL-C and LDL-C, individuals with mutant GG genotype had a higher risk of ACS compared with individuals with CC or CG genotypes (Table 3, OR 1.739, 95% CI: 1.022–2.962; *p* = 0.042). In addition, diabetes, WBC, TC were independent risk factors, while HDL-C was a protective factor for ACS.

**TABLE 3:** Multivariate logistic regression analysis.

	Unadjusted		Adjusted for clinical variables	
	Or (95% CI)	*p* Value	Or (95% CI)	*p* Value
Age	1.002 (0.993–1.012)	0.621	--	
Gender	0.874 (0.722–1.058)	0.168	--	
BMI	0.968 (0.933–1.004)	0.079	--	
Smoking	1.423 (1.183–1.711)	<0.001	1.023 (0.695–1.213)	0.284
Hypertension	1.277 (1.066–1.531)	0.008	1.171 (0.934–1.469);	0.172
Diabetes	2.581 (2.022–3.295)	<0.001	2.263 (1.683–3.044)	<0.001
GG vs CC + CG	1.654 (1.083–2.526)	0.020	1.739 (1.022–2.962)	0.042
WBC	1.443 (1.378–1.512)	<0.001	1.491 (1.413–1.574)	<0.001
TC	1.273 (1.168–1.388)	<0.001	1.227 (1.023–1.472)	0.027
HDL-C	0.307 (0.219–0.431)	<0.001	0.359 (0.241–0.534)	<0.001
LDL-C	1.291 (1.160–1.437)	<0.001	1.084 (0.762–1.301)	0.704

BMI, body mass index; WBC, white blood cells; TC, total cholesterol; HDL-C, high-density lipoprotein cholesterol; LDL-C, low-density lipoprotein cholesterol.

### Angiography Findings and Stent Implant

As PCI will have significant influence in clinical outcomes and prognosis, we analyzed PCI data in ACS patients and the details are presented in Table 4. Of 963 ACS patients, 548 (56.9%) received PCI procedures. Left anterior descending (LAD) artery lesion was more often than lesions in left circumflex (LCX), right coronary (RCA) or left main (LM) artery and 69.6% ACS patients had multivessel diseases (≥2). The majority of ACS patients (78.1%) received one stent implanted. In subgroups of ACS patients with GG genotype compared with CC and CG genotypes, no significant differences were observed in the rate of PCI, culprit artery and the number of diseased artery between two subgroups. Patients with rs2070766 GG genotype had greater gensini score than those with CC and CG genotypes, but it did not reach to statistical significance. Compared to CC and CG genotypes, ACS patients with GG genotype had more stents (≥3) implanted (*p* < 0.001).

**TABLE 4:** Interventional data in ACS patients and recessive model subgroups.

	Total	GG genotype	CC + CG genotype	*p* Value
PCI, n (%)	548 (56.9)	34 (56.7)	514 (56.9)	0.969
LAD lesion, n (%)	796 (82.7)	47 (78.3)	750 (83.0)	0.350
LCX lesion, n (%)	563 (58.5)	33 (55.0)	530 (58.8)	0.567
RCA lesion, n (%)	630 (65.5)	35 (58.3)	595 (65.9)	0.229
LM lesion, n (%)	81 (8.4)	6 (10.0)	76 (8.3)	0.649
Single-vessel disease, n (%)	293 (30.4)	19 (31.7)	274 (30.3)	0.829
Multivessel diseases (≥2), n (%)	670 (69.6)	41 (68.3)	629 (69.7)	
Gensini score	46 (24–81)	50 (30–82)	36 (16–78)	0.510
*Stent number per patient, n (%)*				
1	428 (78.1)	21 (61.7)	407 (79.1)	<0.001
2	102 (18.6)	8 (23.5)	94 (18.3)	
≥3	18 (3.3)	5 (14.7)	13 (2.6)	

Gensini score is expressed as median (25th - 75th percentiles), other values are expressed as number and percentage. ACS, acute coronary artery syndromes; PCI, percutaneous coronary intervention; LAD, left anterior descending artery; LCX, left circumflex artery; RCA, right coronary artery; LM, left main coronary artery.

### Major Adverse Cardiovascular Events During the Follow-Up Period

**FIGURE 2:**
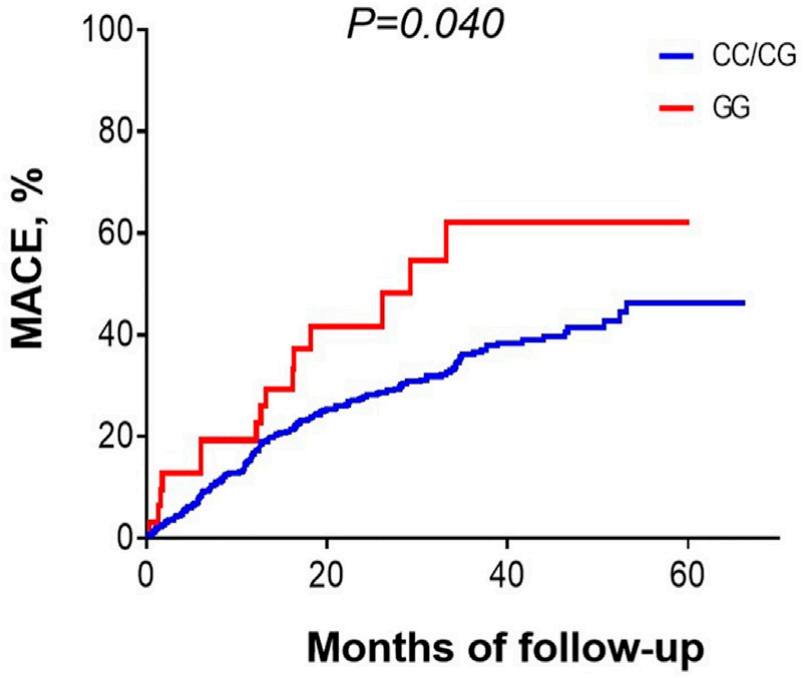
Kaplan-Meier curves showing the prevalence of major adverse cardiovascular events in patients with different *MIF* genotypes during 25 months (12–60 months) follow-up period.

During hospitalization and after discharge, we followed up 548 ACS patients who received PCI to explore the potential influence of *MIF* gene rs2070766 variation in long-term outcomes. During the 25-months (range 12–60 months) follow-up period, 4 (0.7%) patients died in hospital after PCI, 197 patients developed MACE including re-hospitalization owing to recurrent angina or heart failure, target lesion revascularization, cardiac death, non-fatal MI, and stent thrombosis (Table 5). Kaplan-Meier curves showed that the prevalence of MACE was significantly higher in ACS patients carrying GG genotype than those with CC or CG genotypes during the follow-up period (Figure 2, *p* < 0.05).

**TABLE 5:** Major adverse cardiovascular events (MACE) in ACS patients during hospitalization and the 25-months follow-up period after discharge.

MACE (total *n* = 197)	N (%)
Re-hospitalization owing to recurrent angina	87 (44.0)
Re-hospitalization owing to heart failure	39 (20.0)
Target lesion revascularization	33 (17.0)
Cardiac death	22 (11.0)
Non-fatal myocardial infarction	8 (4.0)
Stent thrombosis	8 (4.0)

### Predictive Nomogram for Acute Coronary Syndromes

**FIGURE 3:**
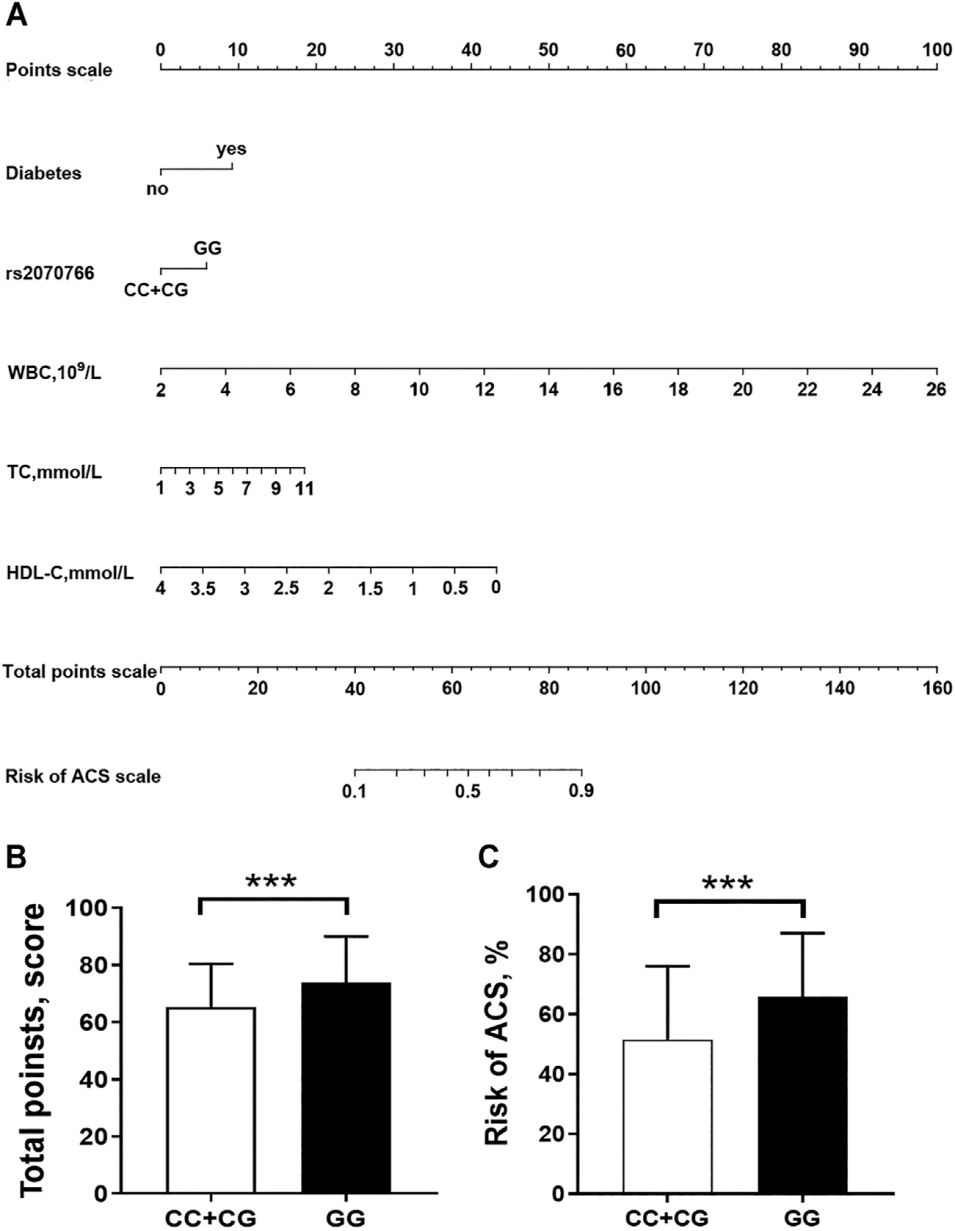
Nomogram to predict the risk of ACS. **(A)** a nomogram was generated by using a number of clinical variables including diabetes, WBC, white blood cell count; TC, total cholesterol; HDL-C, high-density lipoprotein cholesterol. (Figure was created by R software, https://www.r-project.org/). An individual participant value is located on each variable axis, and a line is drawn upward to determine the number of “Points scale” received for each variable value. The sum of these numbers is located on the “Total Points scale” axis to determine the risk of ACS. **(B,C)**, comparisons of the nomogram total points and risk of ACS levels between persons who carrying CC + CG genotypes and GG genotype ***p < 0.0001.

According to the method described in statistical section, using *MIF* rs2070766 genotypes and clinical variables (diabetes, WBC, TC and HDL-C), we developed a nomogram model to predict risk of ACS (Figure 3A). For an example of the clinical utility of the nomogram, a person with diabetes (9.2 points), rs2070766 GG genotype (5.9 points), WBC of 8.9 × 10^9^/L (28.6 points), TC of 5.6 mmol/L (8.2 points), HDL-C of 1.25 mmol/L (30.14 points), nomogram total points scale is 82.04, would have an estimated 85.2% chance of experiencing ACS. In addition, the nomogram total points and risk of ACS levels were significantly higher in individuals with GG genotype than individuals who carrying CC and CG genotypes (Figures 3B,C), the total point of GG genotype carriers was 68.66 (63.63–81.61), CC + CG genotype carriers was 62.37 (55.15–73.15), the GG genotype carriers risk of ACS is 62.20 (50.63–84.74), CC + CG genotype carriers risk of ACS is 47.67 (31.62–71.48), the GG genotype carriers had higher total points and risk of ACS (*p* < 0.05).

### Validation of the Nomogram

**FIGURE 4:**
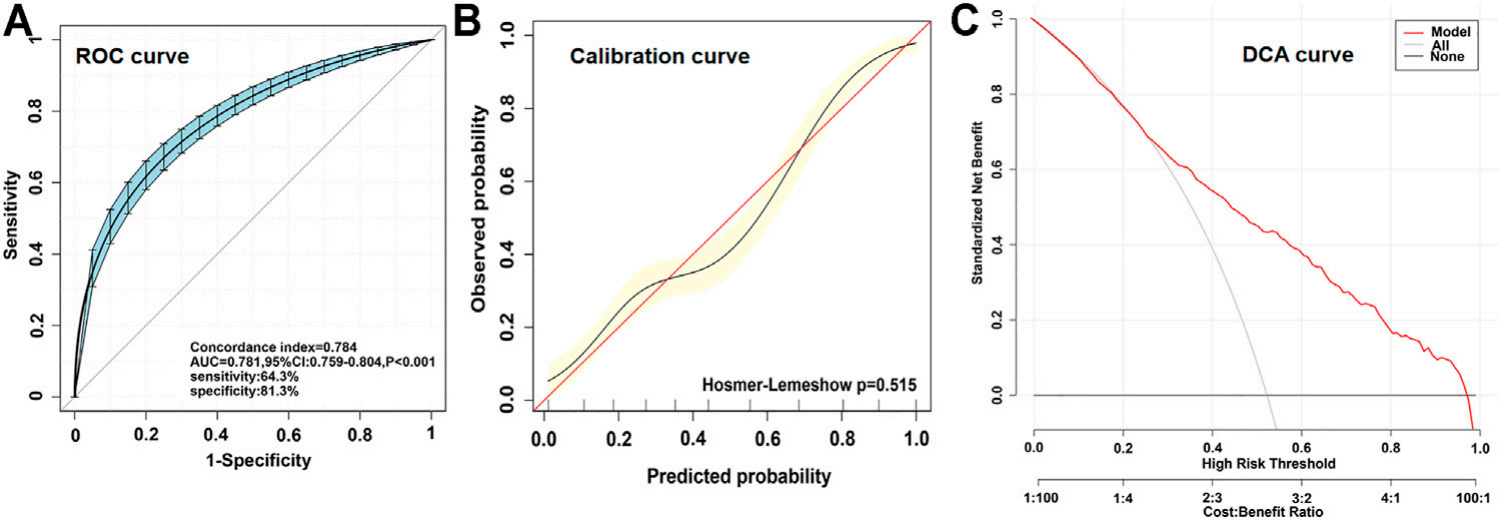
Different parameters to validating the nomogram. **(A)**, receiver operation characteristic curve (ROC) for validating the discrimination power of the nomogram. **(B)**, calibration plot of the nomogram (*p* = 0.515). The diagonal red line represents a perfect prediction by an ideal model. The diagonal 45° red line indicates a perfect calibration that the predictive capability of the model perfectly matches the actual risk of ACS. The black line represents the performance of the nomogram, of which a closer fit to the diagonal red line represents a better prediction. **(C)**, decision curve analysis (DCA) of the nomogram. The *x*-axis indicates the threshold probability. The threshold probability is where the expected benefit of treatment is equal to the expected benefit of avoiding treatment. The *y*-axis measures the net benefit calculated by adding true positives and subtracting false positives. The gray line displays the net benefit of the strategy of treating all ACS patients. The black line illustrates the net benefit of the strategy of treating no ACS patients. The red line indicates the nomogram.

Validation of this nomogram model was based on discrimination, calibration and DCA. This nomogram was validated internally by bootstrap method with 1,000 resamples. This predicting nomogram possessed a good discriminative ability, as shown in Figure 4A, the AUC value was 0.781 (95% CI: 0.759–0.804; *p* < 0.001) and the C-index was 0.784 (95% CI: 0.762–0.806; *p* < 0.001), respectively, indicating the model with good predictive power. The calibration of the predictive model and the calibration curve (Figure 4B) were obtained. In calibration curve of the nomogram model (Figure 4B), the Hosmer-Lemeshow test (*p =* 0.515), demonstrated that the predicted probability was highly consistent with the actual probability. As shown in Figure 4C, the DCA indicated that when the threshold probabilities ranged between 0.30 and 0.95, the use of the nomogram to predict likelihood of ACS risk provided a greater net benefit than the “treat all” or “treat none” strategies, which indicates a clinical usefulness of the nomogram.

## Discussion

ACS is a complex disorder that involves multiple environmental and genetic factors. Compelling evidence from family history and epidemiological studies suggests a genetic basis contributing to the development of ACS (Morgan et al., 2007). Since the traditional environment and lifestyle factors reflects only a small part of mechanisms related to the development of ACS, genetic influence in ACS was not fully addressed. Discovery of genetic risk factors is of great interest in clinical practice, and will help improving the management of ACS. In the present study, we found the frequency of GG variant of *MIF* gene rs2070766 was higher in ACS patients than those with CC or CG genotype. Based on logistic regression analysis and clinical characteristics, a nomogram was developed to help for identifying patients who might develop ACS. Moreover, during the follow-up period of 25 months, ACS patients carrying GG phenotype developed more MACE compared to CC and CG carriers.

MIF is a pleotropic cytokine involving in a variety of inflammatory disorders (Morand et al., 2006; Zernecke et al., 2008). Diverse inflammatory stimuli can trigger MIF secretion to produce pro-inflammatory and pro-atherogenic action (van der Vorst et al., 2015). The role of MIF in the progress of atherosclerosis has drawn intensive attention. Previous studies documented that MIF was produced abundantly by various cells in all types of human atherosclerotic lesions (Noels et al., 2009). MIF enhances oxidized LDL uptake by macrophages (Ayoub et al., 2008). A correlation between the MIF expression and lipid deposition in atherosclerotic plaques indicates an important role in plaque development and lesion progression. Clinical studies found a high MIF level in patients with ACS (Muller et al., 2012) and heart failure (Luedike et al., 2018). White et al. also reported a proinflammatory role of MIF in acute myocardial infarction (White et al., 2013). These results clearly demonstrated an essential role of MIF in the development and progression of atherosclerosis and ischemic heart disease.

Five polymorphisms of the human *MIF* gene have been reported including one 5-8- CATT tetranucleotide repeat at the position of -794 CATT_5-8_ (rs5844572) and four SNPs at positions of -173 G/C (rs755622), +254 T/C (rs2096525), +656 C/G (rs2070766) and rs1007888 (C/T) (Lehmann et al., 2006; White et al., 2013; Valdes-Alvarado et al., 2014; Tilstam et al., 2017; Jankauskas et al., 2019). Rs5844572 and rs755622 are located in the promoter region and rs1007888 located in the 3′ flanking region of the *MIF* gene. The +254 (rs2096525) and +656 (rs2070766) SNPs are located in introns and, thus, do not affect the coding sequence of the *MIF* gene. Albeit the polymorphism of rs2070766 gene is located introns and do not affect the coding sequence of the *MIF* gene, rs2070766 is very close to the third exon, only 5 bases away, may induce splicing mutations, a new splicing site is formed at the mutation point, which affects the expression and function of the protein level (Wang et al., 2015). In the present study, we investigated the relationship between *MIF* gene rs2070766 polymorphism in introns and ACS. In this age and gender matched case-control study, variation of *MIF* gene rs2070766 was classified into three genotypes, CC CG and GG. We found that the detected frequency of rs2070766 GG genotype was significantly higher in ACS patients than in control subjects. When analyzing men and women separately, there was only an association between the SNP rs2070766 recessive model (GG vs CC + CG) and ACS in women, but not in men. After adjusting for other confounders, logistic regression analysis showed a significant difference remained in recessive model (GG vs CC + CG). This result indicates an association between GG genotype of *MIF* gene rs2070766 and risk of ACS. The mechanisms which link the genetic variant of *MIF* to ACS are largely unclear. The possible pathophysiological rationales may be that MIF could increase CAD susceptibility by affecting the metabolism of glycolipid, obesity and inflammation. Herder et al. (2006) found that the elevation of systemic MIF concentrations preceded the onset of type 2 diabetes. Nishihira and Sakaue (2012) found that a tetranucleotide CATT repeat located at position -794 CATT_5-8_ (rs5844572) affects MIF mRNA expression, and is considered to be associated with adiposity. The pro-inflammatory function of MIF has previously been reported in many inflammatory diseases such as arthritis (Calandra and Roger, 2003), septic shock (Bernhagen et al., 1993), colitis and atherosclerosis (Schober et al., 2008). Chemokine-like function of MIF (Schober et al., 2008) and its ability to promote other cytokines production (White et al., 2013) play important role in the evolvement of atherosclerotic lesions.

Nomograms have been shown to be more accurate than conventional systems for predicting outcomes in cardiovascular diseases (Wu et al., 2018). To explore the potential of *MIF* gene variation, we established a nomogram composed of *MIF* rs2070766 genotypes, diabetes, WBC, TC and HDL-C to predict the risk of ACS. In light of the AUC value and C-index >0.7 combined with the calibration plots, the discrimination and calibration capacity of this nomogram model showed good practical values. DCA is a novel method for evaluating prediction models like nomograms (Mo et al., 2018). DCA in this study showed that the nomogram is useful to predict ACS, and is able to visualize the net benefit of clinical consequences according to the threshold probability. Taken together, this nomogram may be able to help cardiologists stratifying patients according to their risk of developing ACS. During the 25 months follow-up, Kaplan-Meier analysis identified that ACS patients carrying GG phenotype developed more MACE than CC or GG phenotype carriers. In another case-control study including 363 CAD patients and 1980 healthy controls, Christian et al. also found that carriers of the minor alleles rs755622 C and rs2070766 G in women had a higher risk of CAD during the 10.3 years follow-up period (Herder et al., 2008). Thus, analysis of certain *MIF* gene polymorphism would help to identify individuals with potential ACS risk, and identification of targeted *MIF* gene variation in patients with ACS may also benefit in risk stratification and management.

There are some limitations in this study. First, this study is a single-center study and the sample size of the study is relative small. Therefore, future studies with larger sample sizes and multi-center cohorts are warranted to validate our results. Second, internal random verification was used for the model validity, the generalisability (external validity) of the study is still unclear. Third, all participants are from China, the findings in this study require further confirmation in other populations.

In conclusion, our results demonstrated an association between the intron genetic variation of *MIF* gene rs2070766 and risk of ACS. The GG genotype carriers of ACS patients had a worse clinical outcome evidenced by a higher incidence of MACE during the follow-up period. We established an early warning model incorporating clinical characteristics and *MIF* gene variation that may be useful as a predictive method to further stratify the risk of ACS patients, which will help for a better management for this emergent event of CAD.

## Data Availability

The authors acknowledge that the data presented in this study must be deposited and made publicly available in an acceptable repository, prior to publication. Frontiers cannot accept a article that does not adhere to our open data policies.

## References

[B1] Adi D. Abuzhalihan J. Wang Y.-h. Baituola G. Wu Y. Xie X. (2020). IDOL Gene Variant Is Associated with Hyperlipidemia in Han Population in Xinjiang, China. Sci. Rep. 10, 14280. 10.1038/s41598-020-71241-1 32868861 PMC7459279

[B2] Ayoub S. Hickey M. J. Morand E. F. (2008). Mechanisms of Disease: Macrophage Migration Inhibitory Factor in SLE, RA and Atherosclerosis. Nat. Rev. Rheumatol. 4, 98–105. 10.1038/ncprheum0701 18235539

[B3] Baugh J. A. Chitnis S. Donnelly S. C. Monteiro J. Lin X. Plant B. J. (2002). A Functional Promoter Polymorphism in the Macrophage Migration Inhibitory Factor (MIF) Gene Associated with Disease Severity in Rheumatoid Arthritis. Genes Immun. 3, 170–176. 10.1038/sj.gene.6363867 12070782

[B4] Bernhagen J. Calandra T. Mitchell R. A. Martin S. B. Tracey K. J. Voelter W. (1993). MIF Is a Pituitary-Derived Cytokine that Potentiates Lethal Endotoxaemia. Nature 365, 756–759. 10.1038/365756a0 8413654

[B5] Boekholdt S. M. Peters R. J. G. Day N. E. Luben R. Bingham S. A. Wareham N. J. (2004). Macrophage Migration Inhibitory Factor and the Risk of Myocardial Infarction or Death Due to Coronary Artery Disease in Adults without Prior Myocardial Infarction or Stroke: the EPIC-Norfolk Prospective Population Study. Am. J. Med. 117, 390–397. 10.1016/j.amjmed.2004.04.010 15380495

[B6] Burger-Kentischer A. Goebel H. Seiler R. Fraedrich G. Schaefer H. E. Dimmeler S. (2002). Expression of Macrophage Migration Inhibitory Factor in Different Stages of Human Atherosclerosis. Circulation 105, 1561–1566. 10.1161/01.cir.0000012942.49244.82 11927523

[B7] Calandra T. Roger T. (2003). Macrophage Migration Inhibitory Factor: a Regulator of Innate Immunity. Nat. Rev. Immunol. 3, 791–800. 10.1038/nri1200 14502271 PMC7097468

[B8] Cannon C. P. Brindis R. G. Chaitman B. R. Cohen D. J. Cross J. T. Jr. Drozda J. P. Jr. (2013). 2013 ACCF/AHA Key Data Elements and Definitions for Measuring the Clinical Management and Outcomes of Patients with Acute Coronary Syndromes and Coronary Artery Disease. Crit. Pathw Cardiol. 12, 65–105. 10.1097/HPC.0b013e3182846e16 23680811

[B9] Chan W. White D. A. Wang X. Y. Bai R. F. Liu Y. Yu H. Y. (2013). Macrophage Migration Inhibitory Factor for the Early Prediction of Infarct Size. Jaha 2, e000226. 10.1161/JAHA.113.000226 24096574 PMC3835222

[B10] Çoban N. Erkan A. F. Ekici B. Kaşit M. Erginel Ünaltuna N. Vurgun E. (2019). Macrophage Migration Inhibitory Factor (MIF) Gene -173 G>C Polymorphism and its Relationship to Coronary Artery Disease and Type 2 Diabetes. Turk Kardiyol Dern Ars 47, 29–37. 10.5543/tkda.2018.35005 30628898

[B11] Du G.-L. Luo J.-Y. Wang D. Li Y.-H. Fang B.-B. Li X.-M. (2020). MIF Gene Rs755622 Polymorphism Positively Associated with Acute Coronary Syndrome in Chinese Han Population: Case-Control Study. Sci. Rep. 10, 140. 10.1038/s41598-019-56949-z 31924846 PMC6954175

[B12] Gao L. Flores C. Fan-Ma S. Miller E. J. Moitra J. Moreno L. (2007). Macrophage Migration Inhibitory Factor in Acute Lung Injury: Expression, Biomarker, and Associations. Transl. Res. 150, 18–29. 10.1016/j.trsl.2007.02.007 17585860 PMC1989118

[B13] Gresele P. Falcinelli E. Loffredo F. Cimmino G. Corazzi T. Forte L. (2011). Platelets Release Matrix Metalloproteinase-2 in the Coronary Circulation of Patients with Acute Coronary Syndromes: Possible Role in Sustained Platelet Activation. Eur. Heart J. 32, 316–325. 10.1093/eurheartj/ehq390 21036774

[B14] Harrell F. E. Jr. Califf R. M. Pryor D. B. Lee K. L. Rosati R. A. (1982). Evaluating the Yield of Medical Tests. JAMA 247, 2543–2546. 10.1001/jama.247.18.2543 7069920

[B15] Heizhati M. Wang L. Yao X. Li M. Hong J. Luo Q. (2020). Prevalence, Awareness, Treatment and Control of Hypertension in Various Ethnic Groups (Hui, Kazakh, Kyrgyz, Mongolian, Tajik) in Xinjiang, Northwest China. Blood Press. 29, 276–284. 10.1080/08037051.2020.1745055 32349556

[B16] Herder C. Kolb H. Koenig W. Haastert B. Muller-Scholze S. Rathmann W. (2006). Association of Systemic Concentrations of Macrophage Migration Inhibitory Factor with Impaired Glucose Tolerance and Type 2 Diabetes: Results from the Cooperative Health Research in the Region of Augsburg, Survey 4 (KORA S4). Diabetes Care 29, 368–371. 10.2337/diacare.29.02.06.dc05-1474 16443889

[B17] Herder C. Illig T. Baumert J. Müller M. Klopp N. Khuseyinova N. (2008). Macrophage Migration Inhibitory Factor (MIF) and Risk for Coronary Heart Disease: Results from the MONICA/KORA Augsburg Case-Cohort Study, 1984-2002. Atherosclerosis 200, 380–388. 10.1016/j.atherosclerosis.2007.12.025 18242614

[B18] Hyde E. K. Martin D. E. Rieger K. L. (2020). Factors Shaping the Provision of Sexual Health Education for Adults with Acute Coronary Syndrome: A Scoping Review. Patient Educ. Couns. 103, 877–887. 10.1016/j.pec.2019.11.017 31767244

[B19] Jankauskas S. S. Wong D. W. L. Bucala R. Djudjaj S. Boor P. (2019). Evolving Complexity of MIF Signaling. Cell Signal. 57, 76–88. 10.1016/j.cellsig.2019.01.006 30682543

[B20] Kerr K. F. Brown M. D. Zhu K. Janes H. (2016). Assessing the Clinical Impact of Risk Prediction Models with Decision Curves: Guidance for Correct Interpretation and Appropriate Use. Jco 34, 2534–2540. 10.1200/JCO.2015.65.5654 PMC4962736 27247223

[B21] Kim S. Schaubel D. E. Mccullough K. P. (2018). A C-Index for Recurrent Event Data: Application to Hospitalizations Among Dialysis Patients. Biom 74, 734–743. 10.1111/biom.12761 PMC6647832 28771674

[B22] Kramer A. A. Zimmerman J. E. (2007). Assessing the Calibration of Mortality Benchmarks in Critical Care: The Hosmer-Lemeshow Test Revisited*. Crit. Care Med. 35, 2052–2056. 10.1097/01.CCM.0000275267.64078.B0 17568333

[B23] Lan M.-Y. Chang Y.-Y. Chen W.-H. Tseng Y.-L. Lin H.-S. Lai S.-L. (2013). Association between MIF Gene Polymorphisms and Carotid Artery Atherosclerosis. Biochem. Biophys. Res. Commun. 435, 319–322. 10.1016/j.bbrc.2013.02.129 23537651

[B24] Lehmann L. Schroeder S. Hartmann W. Dewald O. Book M. Weber S. (2006). A Single Nucleotide Polymorphism of Macrophage Migration Inhibitory Factor Is Related to Inflammatory Response in Coronary Bypass Surgery Using Cardiopulmonary Bypass. Eur. J. Cardio-Thoracic Surg. 30, 59–63. 10.1016/j.ejcts.2006.01.058 16527487

[B25] Li D.-Y. Zhang J.-Y. Chen Q.-J. Liu F. Zhao Q. Gao X.-M. (2020). MIF -173G/C (Rs755622) Polymorphism Modulates Coronary Artery Disease Risk: Evidence from a Systematic Meta-Analysis. BMC Cardiovasc. Disord. 20, 300. 10.1186/s12872-020-01564-4 32560699 PMC7304150

[B26] Luedike P. Alatzides G. Papathanasiou M. Heisler M. Pohl J. Lehmann N. (2018). Circulating Macrophage Migration Inhibitory Factor (MIF) in Patients with Heart Failure. Cytokine 110, 104–109. 10.1016/j.cyto.2018.04.033 29723777

[B27] Luo J.-Y. Xu R. Li X.-M. Zhou Y. Zhao Q. Liu F. (2016). MIF Gene Polymorphism Rs755622 Is Associated with Coronary Artery Disease and Severity of Coronary Lesions in a Chinese Kazakh Population. Medicine (Baltimore) 95, e2617. 10.1097/MD.0000000000002617 26825917 PMC5291587

[B28] Makino A. Nakamura T. Hirano M. Kitta Y. Sano K. Kobayashi T. (2010). High Plasma Levels of Macrophage Migration Inhibitory Factor Are Associated with Adverse Long-Term Outcome in Patients with Stable Coronary Artery Disease and Impaired Glucose Tolerance or Type 2 Diabetes Mellitus. Atherosclerosis 213, 573–578. 10.1016/j.atherosclerosis.2010.09.004 20934703

[B29] Mason P. J. Shah B. Tamis-Holland J. E. Bittl J. A. Cohen M. G. Safirstein J. (2018). An Update on Radial Artery Access and Best Practices for Transradial Coronary Angiography and Intervention in Acute Coronary Syndrome: A Scientific Statement from the American Heart Association. Circ. Cardiovasc. Interventions 11, e000035. 10.1161/HCV.0000000000000035 30354598

[B30] Mo S. Dai W. Xiang W. Li Q. Wang R. Cai G. (2018). Predictive Factors of Synchronous Colorectal Peritoneal Metastases: Development of a Nomogram and Study of its Utilities Using Decision Curve Analysis. Int. J. Surg. 54, 149–155. 10.1016/j.ijsu.2018.04.051 29730071

[B31] Morand E. F. Leech M. Bernhagen J. (2006). MIF: a New Cytokine Link between Rheumatoid Arthritis and Atherosclerosis. Nat. Rev. Drug Discov. 5, 399–411. 10.1038/nrd2029 16628200

[B32] Morgan T. M. Krumholz H. M. Lifton R. P. Spertus J. A. (2007). Nonvalidation of Reported Genetic Risk Factors for Acute Coronary Syndrome in a Large-Scale Replication Study. JAMA 297, 1551–1561. 10.1001/jama.297.14.1551 17426274

[B33] Müller I. I. Müller K. A. L. Schönleber H. Karathanos A. Schneider M. Jorbenadze R. (2012). Macrophage Migration Inhibitory Factor Is Enhanced in Acute Coronary Syndromes and Is Associated with the Inflammatory Response. PLoS One 7, e38376. 10.1371/journal.pone.0038376 22693633 PMC3367911

[B34] Nishihira J. Sakaue S. (2012). Overview of Macrophage Migration Inhibitory Factor (MIF) as a Potential Biomarker Relevant to Adiposity. J. Tradit. Complement. Med. 2, 186–191. 10.1016/s2225-4110(16)30098-0 24716131 PMC3942894

[B35] Noels H. Bernhagen J. Weber C. (2009). Macrophage Migration Inhibitory Factor: a Noncanonical Chemokine Important in Atherosclerosis. Trends Cardiovasc. Med. 19, 76–86. 10.1016/j.tcm.2009.05.002 19679264

[B36] Pan J.-H. Sukhova G. K. Yang J.-T. Wang B. Xie T. Fu H. (2004). Macrophage Migration Inhibitory Factor Deficiency Impairs Atherosclerosis in Low-Density Lipoprotein Receptor-Deficient Mice. Circulation 109, 3149–3153. 10.1161/01.CIR.0000134704.84454.D2 15197138

[B37] Pan S. Yu Z.-X. Ma Y.-T. Liu F. Yang Y.-N. Ma X. (2013). Appropriate Body Mass index and Waist Circumference Cutoffs for Categorization of Overweight and central Adiposity Among Uighur Adults in Xinjiang. PLoS One 8, e80185. 10.1371/journal.pone.0080185 24244645 PMC3820640

[B38] Radstake T. R. D. J. Sweep F. C. G. J. Welsing P. Franke B. Vermeulen S. H. H. M. Geurts-Moespot A. (2005). Correlation of Rheumatoid Arthritis Severity with the Genetic Functional Variants and Circulating Levels of Macrophage Migration Inhibitory Factor. Arthritis Rheum. 52, 3020–3029. 10.1002/art.21285 16200611

[B39] Roberts R. Campillo A. (2018). Genetic Stratification for Primary Prevention of Coronary Artery Disease. Curr. Opin. Cardiol. 33, 529–534. 10.1097/HCO.0000000000000542 29979201

[B40] Schmeisser A. Marquetant R. Illmer T. Graffy C. Garlichs C. D. Böckler D. (2005). The Expression of Macrophage Migration Inhibitory Factor 1α (MIF 1α) in Human Atherosclerotic Plaques Is Induced by Different Proatherogenic Stimuli and Associated with Plaque Instability. Atherosclerosis 178, 83–94. 10.1016/j.atherosclerosis.2004.08.038 15585204

[B41] Schober A. Bernhagen J. Weber C. (2008). Chemokine-like Functions of MIF in Atherosclerosis. J. Mol. Med. 86, 761–770. 10.1007/s00109-008-0334-2 18385967

[B42] Schomaker M. Heumann C. (2018). Bootstrap Inference when Using Multiple Imputation. Stat. Med. 37, 2252–2266. 10.1002/sim.7654 29682776 PMC5986623

[B43] Sinitski D. Kontos C. Krammer C. Asare Y. Kapurniotu A. Bernhagen J. (2019). Macrophage Migration Inhibitory Factor (MIF)-Based Therapeutic Concepts in Atherosclerosis and Inflammation. Thromb. Haemost. 119, 553–566. 10.1055/s-0039-1677803 30716779

[B44] Tereshchenko I. P. Petrkova J. Mrazek F. Lukl J. Maksimov V. N. Romaschenko A. G. (2009). The Macrophage Migration Inhibitory Factor (MIF) Gene Polymorphism in Czech and Russian Patients with Myocardial Infarction. Clin. Chim. Acta 402, 199–202. 10.1016/j.cca.2008.12.034 19167373

[B45] Tilstam P. V. Qi D. Leng L. Young L. Bucala R. (2017). MIF Family Cytokines in Cardiovascular Diseases and Prospects for Precision-Based Therapeutics. Expert Opin. Ther. Targets 21, 671–683. 10.1080/14728222.2017.1336227 28562118 PMC6130320

[B46] Valdés-Alvarado E. Muñoz-Valle J. F. Valle Y. Sandoval-Pinto E. García-González I. J. Valdez-Haro A. (2014). Association between the −794 (CATT)5-8 MIFGene Polymorphism and Susceptibility to Acute Coronary Syndrome in a Western Mexican Population. J. Immunol. Res. 2014, 1–5. 10.1155/2014/704854 PMC4106097 25105152

[B47] van der Vorst E. P. C. Döring Y. Weber C. (2015). MIF and CXCL12 in Cardiovascular Diseases: Functional Differences and Similarities. Front. Immunol. 6, 373. 10.3389/fimmu.2015.00373 26257740 PMC4508925

[B48] Wang X. Zhao X. Wang X. Yao J. Zhang F. Lang Y. (2015). Two Novel HOGA1 Splicing Mutations Identified in a Chinese Patient with Primary Hyperoxaluria Type 3. Am. J. Nephrol. 42, 78–84. 10.1159/000439232 26340091

[B49] White D. A. Fang L. Chan W. Morand E. F. Kiriazis H. Duffy S. J. (2013). Pro-inflammatory Action of MIF in Acute Myocardial Infarction via Activation of Peripheral Blood Mononuclear Cells. PLoS One 8, e76206. 10.1371/journal.pone.0076206 24098445 PMC3788072

[B50] Wu N. Chen X. Li M. Qu X. Li Y. Xie W. (2018). Predicting Obstructive Coronary Artery Disease Using Carotid Ultrasound Parameters: A Nomogram from a Large Real-World Clinical Data. Eur. J. Clin. Invest. 48, e12956. 10.1111/eci.12956 29782650

[B51] Zernecke A. Bernhagen J. Weber C. (2008). Macrophage Migration Inhibitory Factor in Cardiovascular Disease. Circulation 117, 1594–1602. 10.1161/CIRCULATIONAHA.107.729125 18362243

[B52] Zhao Q. Men L. Li X.-M. Liu F. Shan C.-F. Zhou X.-R. (2019). Circulating MIF Levels Predict Clinical Outcomes in Patients with ST-Elevation Myocardial Infarction after Percutaneous Coronary Intervention. Can. J. Cardiol. 35, 1366–1376. 10.1016/j.cjca.2019.04.028 31495686

[B53] Zheng Q. Zhang Y. Jiang J. Jia J. Fan F. Gong Y. (2020). Exome-Wide Association Study Reveals Several Susceptibility Genes and Pathways Associated with Acute Coronary Syndromes in Han Chinese. Front. Genet. 11, 336. 10.3389/fgene.2020.00336 32328087 PMC7160370

[B54] Zhou Y. He Y. Yang H. Yu H. Wang T. Chen Z. (2020). Development and Validation a Nomogram for Predicting the Risk of Severe COVID-19: A Multi-Center Study in Sichuan, China. PLoS One 15, e0233328. 10.1371/journal.pone.0233328 32421703 PMC7233581

