# Supplementary material for: Genetic Variation of Migration Inhibitory Factor Gene rs2070766 Is Associated With Acute Coronary Syndromes in Chinese Population
Source: Front Genet. 2022 Jan 3;12:750975. doi: 10.3389/fgene.2021.750975 (PMC8762351; doi:10.3389/fgene.2021.750975)
Supplement: Supplementary file 1 [file Table1.DOCX]

**Frontiers in Genetics—750975R1**

**Supplementary materials**

**Genetic variation of** ***MIF*** **gene rs2070766 is associated with acute coronary syndromes in Chinese population**

Jin-Yu Zhang*, Qian Zhao*, Fen Liu, De-Yang Li, Li Men, Jun-Yi Luo, Ling Zhao, Xiao-Mei Li#, Xiao-Ming Gao#, Yi-Ning Yang#

**DETAILED RESULTS**

**Predictive Nomogram for ACS**

Using *MIF* rs2070766 genotypes and clinical variables (diabetes, WBC, TC and HDL-C), we developed a nomogram model to predict risk of ACS. As shown in Figure 3A, this nomogram was used to quantitatively predict the risk probability of ACS. In detail, using the patient’s diabetes (yes/no), a vertical line can be drawn from the variable to the points scale (top) to get a corresponding value. After repeating the process for each variable, the scores for each variable were added up and pointed to a corresponding value on the “Total Points scale” axis (bottom). Finally, a vertical line of the corresponding value on the “Total Points scale” axis can be drawn straight down to the risk of ACS scale axis to evaluate the probability of ACS risk. For an example of the clinical utility of the nomogram, a person with diabetes (9.2 points), rs2070766 GG genotype (5.9 points), WBC of 8.9 × 10^9^/L (28.6 points), TC of 5.6 mmol/L (8.2 points), HDL-C of 1.25 mmol/L (30.14 points), nomogram total points scale is 82.04, would have an estimated 85.2% chance of experiencing ACS. In addition, the nomogram total points and risk of ACS levels were significantly higher in individuals with GG genotype than individuals who carrying CC and CG genotypes (Figure 3B, 3C), the total point of GG genotype carriers was 68.66 (63.63-81.61), CC+CG genotype carriers was 62.37 (55.15-73.15), the GG genotype carriers risk of ACS is 62.20 (50.63-84.74), CC+CG genotype carriers risk of ACS is 47.67 (31.62-71.48), the GG genotype carriers had higher total points and risk of ACS (*P* < 0.05).

**Validation of the Nomogram**

Validation of this nomogram model was based on discrimination, calibration and DCA. This nomogram was validated internally by bootstrap method with 1000 resamples. This predicting nomogram possessed a good discriminative ability, as shown in Figure 4A, the AUC value was 0.781 (95% CI: 0.759-0.804; *P* < 0.001) and the C-index was 0.784 (95% CI: 0.762-0.806; *P* < 0.001), respectively, indicating the model with good predictive power. The calibration of the predictive model was also evaluated by the Hosmer-Lemeshow test, and the calibration curve (Figure 4B) was obtained. When *P* > 0.05, the calibration ability of this model is good. The calibration curve of the nomogram model was *P* = 0.515, it was greater than 0.05, indicating that this model had a good calibration ability. The Hosmer-Lemeshow test result demonstrated that the predicted probability was highly consistent with the actual probability. Moreover, DCA was used to estimate the net benefit of a model based on the difference between the number of true and false-positive results and it is widely used in assessing whether the nomogram-assisted decision would improve patient outcome. As shown in Figure 4C, the DCA indicated that when the threshold probabilities ranged between 0.30 and 0.95, the use of the nomogram to predict likelihood of ACS risk provided a greater net benefit than the “treat all” or “treat none” strategies, which indicates a clinical usefulness of the nomogram. Zero net benefit in the DCA represents that no patients with ACS require further treatment. ACS patients with an elevated probability may possibly need further therapy, such as PCI or additional drugs. The decision curve showed that if the threshold probability is between 0.30 and 0.95, then using this nomogram to predict ACS adds more benefit than treating either all or no patients. For example, if the threshold probability of a patient is 60% (the patient would opt for treatment if the probability of ACS was > 40%), then the net benefit is 0.4.
